# Evolution in the Use of Statistical Testing in the Field of Orthopedics From 1993 to 2023

**DOI:** 10.7759/cureus.72709

**Published:** 2024-10-30

**Authors:** Ting D Zhang, Mila Scheinberg, Alexander Hoffman, Labdhi Mehta, Caleb Hayes, Samuel Schick, Marc Bernstein, Ashish Shah

**Affiliations:** 1 Orthopedic Surgery, University of Alabama at Birmingham, Birmingham, USA; 2 Orthopedics, University of Alabama at Birmingham, Birmingham, USA

**Keywords:** medical education, orthopedic literature, orthopedic subspecialties, power analysis, publication trends, statistical test

## Abstract

Statistical tests are important tools in research as they provide a systematic and objective approach to analyzing data, testing hypotheses, and drawing conclusions. We investigated the application of statistical tests used in articles published in the Journal of Bone and Joint Surgery (JBJS) over the last 30 years. We searched PubMed for JBJS articles published from 1993 to 2023 and randomly sampled 5% of articles from each year. Our inclusion criteria identified a selection of articles for review, and we collected and analyzed variables, including statements of significance, significance level, power calculations, usage of statistical tests and methods, power analysis, and orthopedic subspecialties. Articles were grouped into three 10-year periods, and then analysis of variance, Tukey’s honestly significant difference, and chi-square tests were conducted to investigate the changes between decade intervals. Our 5% stratified random sample of JBJS publications yielded 593 unique articles, of which 559 were accessible as full-text articles. Common statistical tests included t-test, chi-squared, and ANOVA. The average number of statistical tests per paper increased from 1993-2002 (n = 0.64) to 2013-2023 (n = 1.25) (p value = 0.0009). Among the articles stating alpha levels (n = 156), 0.05 was most prevalent (94%), with 0.01 and 0.1 less commonly used (3.2% and 1.2%, respectively). Article types varied, with "commentary, editor, forum" being the most common (n = 124), followed by retrospective cohort studies (n = 105). The most frequent power goals were 80% and 90%. In studies reporting power calculations, 34% declared adequate power, 14% admitted insufficient power, and 52% did not disclose adequacy. Statistical test and publication trends varied greatly between orthopedic subspecialties. With the increasing usage of statistical tests in orthopedic research, it is increasingly important for readers to have a strong understanding of statistical analysis. This foundational knowledge will enable critical engagement with literature and aid in the growth of the orthopedic field.

## Introduction and background

Statistical tests play a crucial role in research by providing a framework for making inferences and drawing conclusions from data [1]. They are usually beneficial and help researchers determine whether the patterns or differences observed in their data are statistically significant or simply due to chance. However, these test results are often subject to reporting biases and may mislead readers or mask shortcomings in the research’s experimental design [2,3]. Thus, researchers and readers must have at least a basic understanding of how statistical analyses are conducted. While research is becoming increasingly complex, the complexity of statistical tests may not necessarily increase in direct proportion [4]. The application of statistical methods and tests continues to evolve to address the increasing intricacies of research questions. However, there is a paucity of literature identifying this evolution. Therefore, our study aims to review and identify trends in statistical test usage in one of the most impactful journals in orthopedic surgery, the Journal of Bone and Joint Surgery (JBJS). Through our review, we identify the most commonly used statistical tests in this journal and discuss how usage trends have changed over the past 30 years. By identifying these trends, this article can serve as a resource for future authors considering the type and complexity of statistical tests to use when publishing in a high-impact orthopedic journal.

## Review

Materials and methods

The data collection process was divided into three steps. First, articles from three 10-year periods were selected and filtered. Second, data were extracted from the filtered articles. Finally, statistical analysis was conducted on the collected data.

Article Selection and Filtering

**Figure 1:**
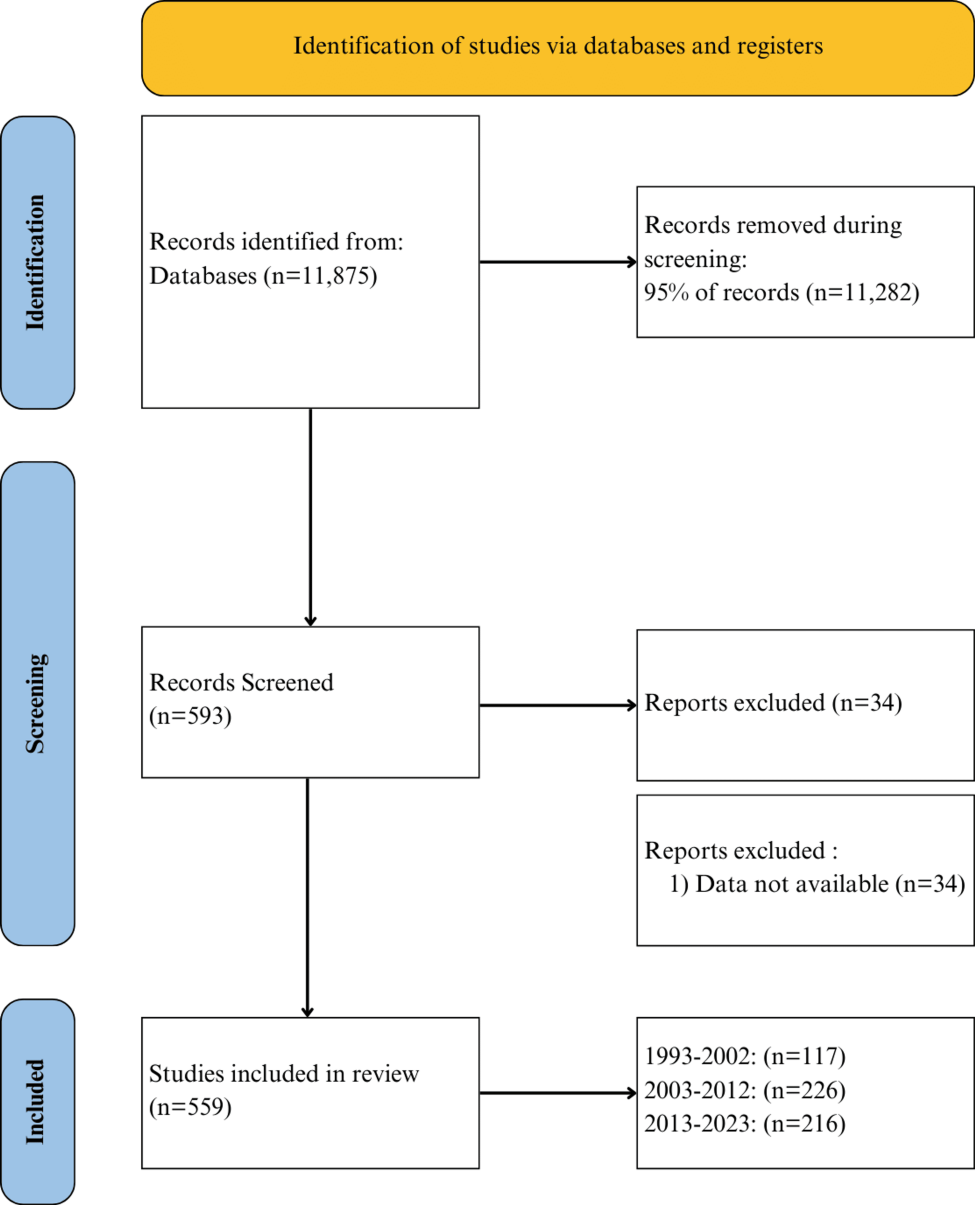
PRISMA Diagram of the Screening Process PRISMA: Preferred Reporting Items for Systematic Reviews and Meta-Analyses

Using the PubMed database, a search was run for all JBJS articles published between January 1, 1993, and July 1, 2023. We selected JBJS as our journal of interest because of its distinguished reputation for high credibility and a notable impact factor. This search yielded 11,875 articles and contained article types including, but not limited to, chart reviews, meta-analyses, randomized controlled trials, systematic reviews, and retrospective cohort studies. A random sampling of this article set stratified by year was then performed, retrieving 5% of all JBJS articles published each year. The 5% stratified random sampling was chosen to allow for an appropriate sample size for ANOVA testing and to ensure an adequate representation of articles per year. By performing a 5% sampling, we defined a sample size large enough to provide sufficient statistical power while maintaining a manageable workload. After sampling, the resulting articles were subject to further screening. Articles were excluded if they represented errata corrections or if no data were available for the article. Direct, manual screening was also completed, which yielded the final article set selected for testing. Figure 1 shows a detailed Preferred Reporting Items for Systematic Reviews and Meta-Analyses (PRISMA) diagram of our selection process.

Data Collection

Three research fellows (AH, CH, and LM) independently reviewed the articles and extracted data. Information was collected regarding authors, publishing date, statements of significance, significance level, power calculations, usage of statistical tests and methods, power analysis, and orthopedic subspecialties. Article type was also noted based on study design. Nonexperimental articles were grouped into categories of commentary, editorials, and forums. The totals for article type and statistical tests utilized were tallied. Only inferential statistical tests were included in the analysis, and descriptive statistics, such as mean, median, and range, were not counted. Two reviewers (MS and TZ) confirmed the accuracy of data collection and resolved discrepancies through open discussion or consulting the project investigator if necessary.

Statistical Analysis

One-way ANOVA testing was conducted, with time intervals defined as the independent variable and the number of statistical tests per article as the dependent variable. Time intervals were split into three groups: 1993-2002 (A), 2003-2012 (B), and 2013-2023 (C). Statistical significance was determined by F-statistic and p value data, and Tukey’s honestly significant difference (HSD) testing provided pairwise comparisons between time intervals for further analysis. Chi-square testing was also conducted to compare the presence of power analysis in articles over time. The alpha value for all tests was 0.05 based on the conventionally acceptable probability of making a type I error [5].

Results

In total, 593 articles were sampled from 1993 to 2023. Of these articles, data were available for 559 articles. Group A included 117 articles, Group B included 226 articles, and Group C included 216 articles. ANOVA testing of these 559 articles for time intervals and the number of statistical tests per article yielded a statistically significant P value of 0.0026. The mean number of statistical tests per article was 0.64, 0.97, and 1.25 for time intervals A, B, and C, respectively (Table 1). This increase in the number of statistical tests per article was independent of sample size, as the number of articles published increased from time A to B, but then decreased in C. Tukey’s HSD test results between A and C were statistically significant (p value = 0.0009), although pairs A-B and B-C were statistically insignificant (p values = 0.1161, 0.2243, respectively) (Table 2).

**Table 1:** ANOVA Test Results for the Number of Statistical Tests Per Article by Years

	Time Interval		
	A	B	C
N	117	226	216
Mean	0.64	0.97	1.25
Standard deviation	1.09	1.49	1.79
F-ratio	6.01		
P value	0.0026		

**Table 2:** Tukey’s Honestly Significant Difference Test Results M_A_: Mean number of statistical tests per article in time interval A M_B_: Mean number of statistical tests per article in time interval B M_C_: Mean number of statistical tests per article in time interval C

Pairwise Comparisons		Difference of Means	Q-value (P Value)
A:B	M_A_ = 0.64	0.33	Q = 2.81 (p = 0.1161)
	M_B_ = 0.97		
A:C	M_A_ = 0.64	0.61	Q = 5.15 (p = 0.0009)
	M_C_ = 1.25		
B:C	M_B_ = 0.97	0.28	Q = 2.34 (p = 0.2243)
	M_C_ = 1.25		

**Figure 2:**
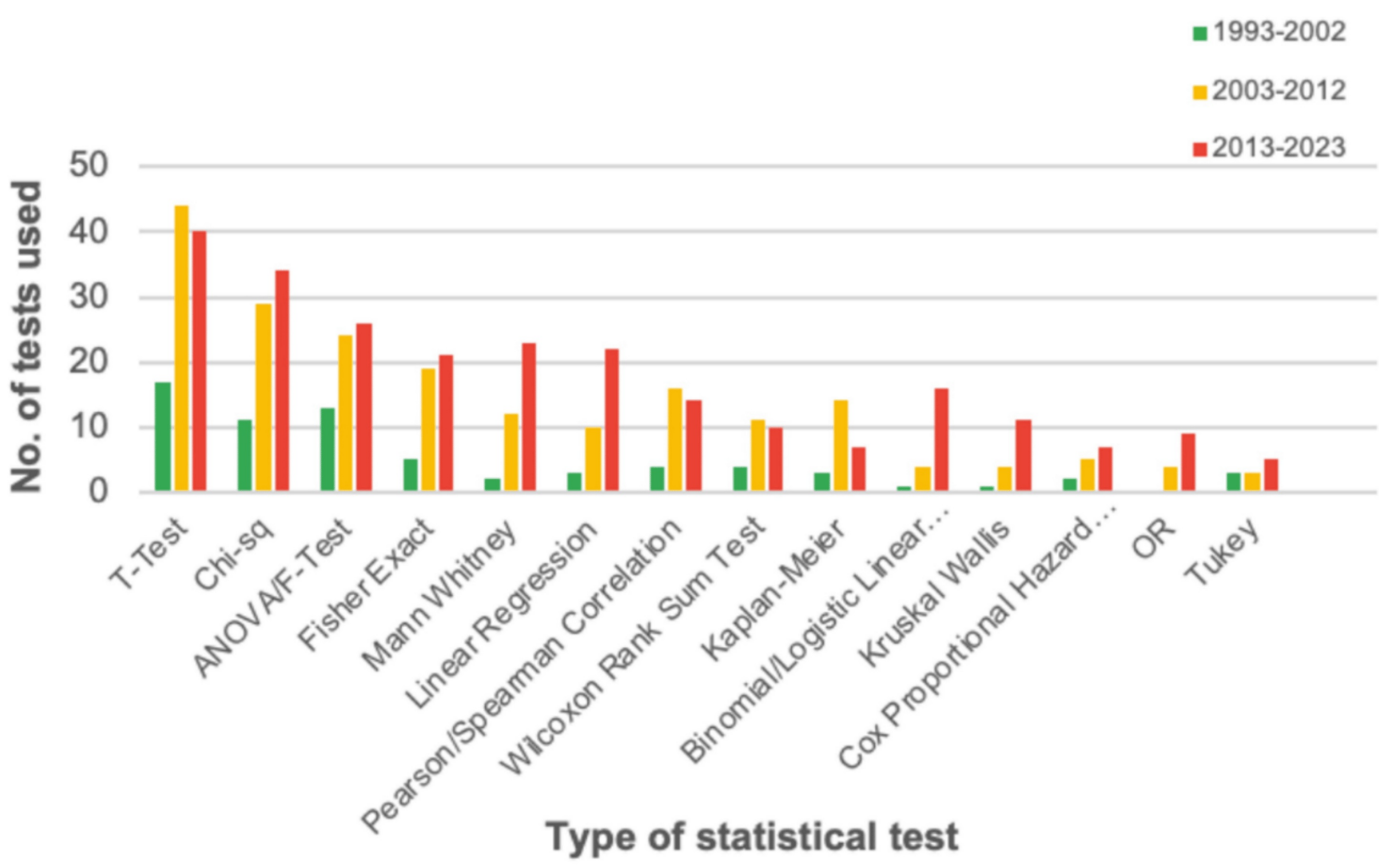
Frequency of the Most Common Statistical Tests Used by Decade

The nearly all statistical tests stud statistical tests and methods used in JBJS articles were, in descending order, the t-test (n = 101), chi-square (n = 74), ANOVA (n = 63), Fisher's exact test (n = 45), Mann-Whitney test (n = 37), linear regression (n = 35), Pearson correlation (n = 34), Kaplan-Meier survival curves (n = 24), and binomial regression (n = 21) (Table 3). Less commonly utilized tests were the Wilcoxon rank sum test (n = 17), Kruskal Wallis (n = 16), Cox proportional hazard (n = 14), odds ratio (n = 13), and Tukey multiple comparisons (n = 11) (Table 3). Statistical test usage is highlighted in Figure 2, and nearly all statistical tests studied experienced an increase in usage over time. From 1993 to 2023, the overall most common article type (n = 124) was the non-investigative category of “commentary, editor, forum,” which includes expert opinion, topic reviews, and updates. The second most commonly published article type was the retrospective cohort study (n = 105), followed by randomized controlled trials (n = 63), biomechanical and basic science studies (n = 57), and prospective cohort studies (n = 51) (Table 4).

**Table 3:** Frequency of the Statistical Tests Used in JBJS JBJS: Journal of Bone and Joint Surgery

Type of Test	n
T-test	101
Chi-square	74
ANOVA/F-test	63
Fisher's exact test	45
Mann-Whitney U test	37
Linear regression	35
Pearson/Spearman correlation	34
Kaplan-Meier estimator	24
Binomial/Logistic linear regression	21
Wilcoxon rank sum test	17
Kruskal-Wallis test	16
Cox proportional hazard regression	14
Odds ratio	13
Tukey	11
Wilcoxon signed rank	8
Log-rank test	6
Shapiro-Wilk test	6
Kolmogorov-Smirnov test	6
McNemar's test	2
Random effects model	2
Z-test	2
Fisher's least significant difference	2
Stepwise linear regression	2
Poisson	1
Hosmer-Lemeshow test	1
Analysis of covariance	1
Cochran's Q test	1
Cochran-Armitage test	1
Holm-Sidak test	1
Levene's median test	1
Likelihood ratio test	1
Logarithmic curve	1
Markov modeling	1
Newman-Keuls method	1
RR test	1

**Table 4:** Frequency of Article Types in JBJS JBJS: The Journal of Bone & Joint Surgery

Article Type	n
Commentary, Editorial, Forum	124
Retrospective Cohort	105
RCT	63
Biomechanical	57
Case Report	52
Prospective Cohort	51
Quality Improvement	36
Systematic Review	26
Case Control	13
Surgical Technique	11
Epidemiological	10
Case Series	5
Survey	2
Case Report	1
Simulation	1
Chart Review	1
Matched Cohort	1

Thresholds for significance were declared in 27.9% (n = 156) of sampled articles. A significance level of 0.05 was the most commonly stated, found in 95.6% of articles declaring significance. Less commonly stated significance thresholds utilized were 0.01 and 0.1, mentioned in 3.1% and 1.9% of articles declaring significance, respectively. Across time intervals A, B, and C, the percentage of articles reporting power increased from 0.04, 0.06, to 0.13 (p = 0.0104). The most common power goal reported was 80% (n = 23), followed by 90% (n = 7). In total, 40 out of 559 articles analyzed reported percentage power, with eight additional articles mentioning power but not reporting percentage. In studies reporting power calculations, 31% explicitly stated that power was adequate, 21% admitted falling short of power requirements, and 48% of studies did not disclose whether power was adequate.

**Figure 3:**
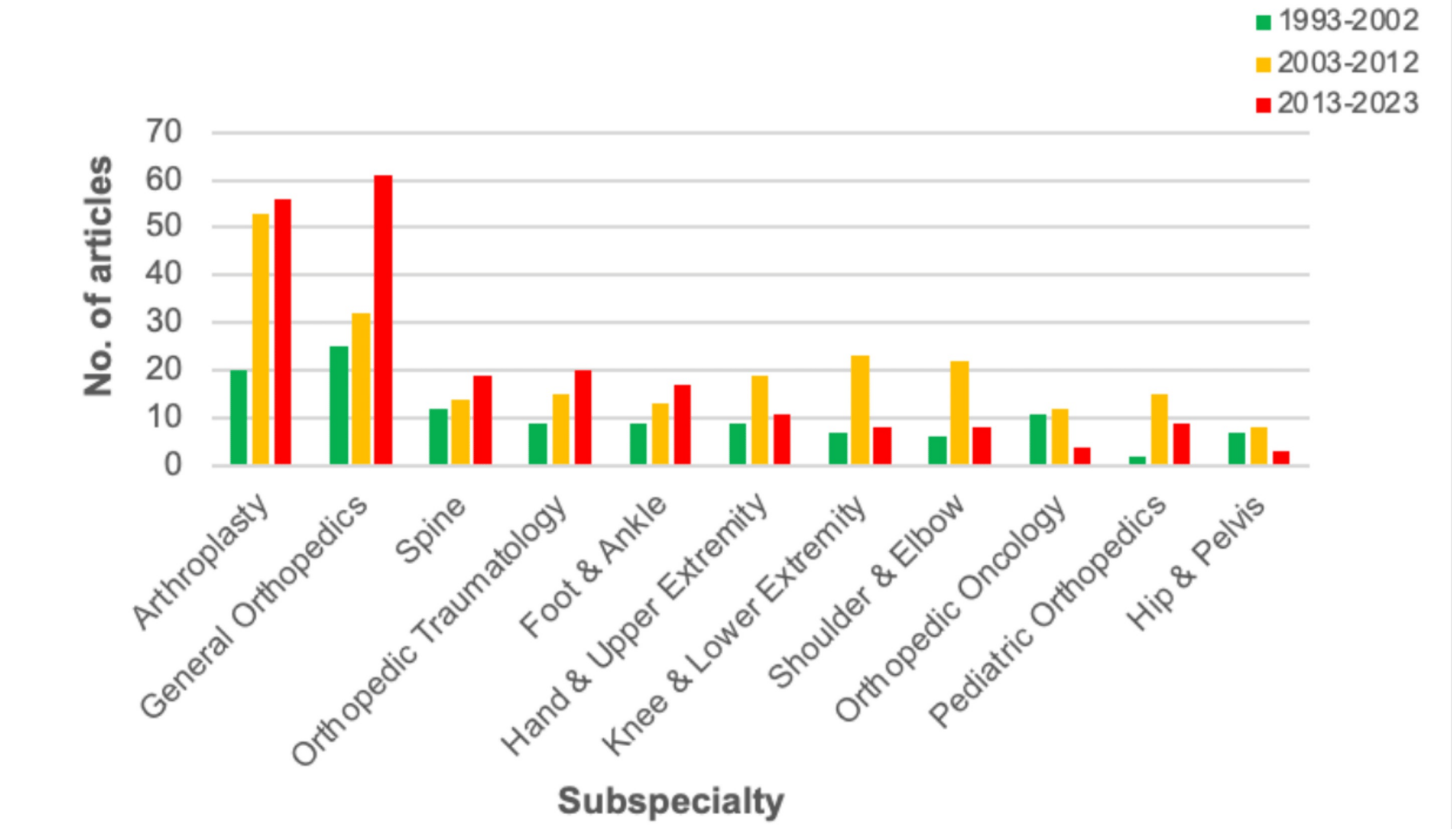
Total Number of Articles Published Over Time by Subspecialty

**Figure 4:**
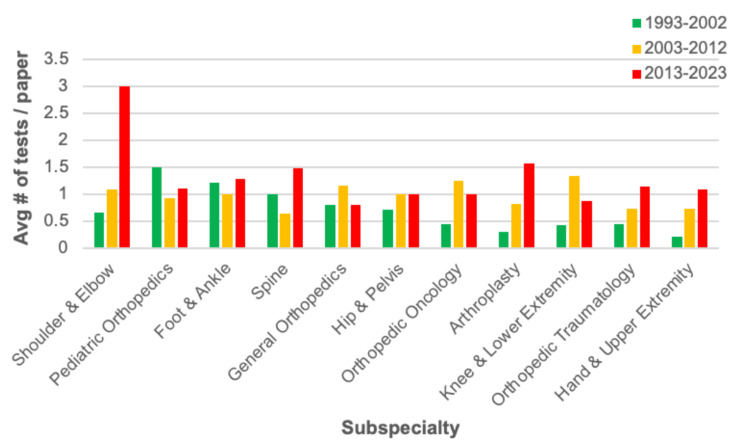
Average Number of Tests Per Paper by Year and Subspecialty

Out of all subspecialties of interest, general orthopedics and arthroplasty had the greatest number of publications, with 20 or more publications recorded in the sample for the 10-year time intervals (Figure 3). Most subspecialties had the lowest number of publications during the 1993-2002 interval, with increases in the 2003-2012 and 2013-2023 periods. Figure 4 illustrates the average number of statistical tests per paper by subspecialty and time, with shoulder and elbow using the highest average number of tests across the 30-year period.

Discussion

The dynamic nature of statistical testing within orthopedic research reflects the changing nature of medical inquiry. Over the years, the spectrum of statistical tests employed has expanded significantly. This development accommodates both the increasing complexity of research questions in the field and the shifts in research focuses, such as the recent emphasis on patient-reported outcomes [6,7]. The emergence of tests such as the t-test, chi-square, ANOVA, and Fisher's exact test as the most common tools underlines their versatility in addressing diverse orthopedic hypotheses. Statistical testing in orthopedic literature has also diversified and increased in use over the years, as seen in the roughly linear increase in the number of statistical tests per article over the three decades studied. Tukey’s HSD testing results also indicate that, despite consistent increases in the number of statistical tests per article per decade, increases are only statistically significant between periods A and C. These results provide a framework for future research regarding orthopedic statistical test usage; time intervals studied should span a minimum of a few decades to best identify trends.

The distribution of article types in the JBJS highlights the multifaceted nature of orthopedic research. The prevalence of "commentary, editor, forum" articles highlights the prominent role of expert opinions and discussions in shaping orthopedic discourse [8]. Such articles contribute to integrating research findings into clinical practice, bridging the gap between evidence and application. The prominence of retrospective cohort studies suggests the enduring value of historical data in orthopedic investigations [9]. This study type, often utilized to examine long-term outcomes and trends, underscores the significance of retrospective analyses in guiding contemporary orthopedic practices. Compared to randomized clinical trial data, evidence from these studies is of lower quality, but the threshold for accruing data for analysis is lower [10]. The authors of this paper encourage the usage of high-quality evidence from RCTs and meta-analyses of RCTs in clinical decision-making where possible.

The prevalence of the 0.05 significance level in most articles underscores the traditional threshold for establishing statistical significance that traces its origins back to preeminent statistician Ronald Fisher proposing the use of convenient cutoffs in his 1925 book “Statistical Methods for Research Workers” [11]. Although initially intended as a suggestion and despite debate within the research community, Fisher’s 0.05 significance level remains the most common alpha used in statistical testing [12,13]. This value maintains an acceptable probability of committing a type I error and sufficient statistical power [5,14]. The presence of varying significance levels, such as 0.01 and 0.1, reveals researchers' nuanced approach in orthopedics [6]. The choice of the significance level is not arbitrary; it is influenced by factors such as the clinical context, potential consequences of type I and type II errors, and overall risk-benefit assessment [15-17]. As orthopedic interventions directly affect patient care, selecting significance levels depends on clinical contexts and is vital in ensuring accurate decision-making [14]. 

The reported power considerations within the analyzed articles provide insights into the rigor of statistical analyses in orthopedic research. Power is the probability of falsely rejecting the null hypothesis and is calculated as type II error probability [18]. The prominence of 80% and 90% power goals aligns with established conventions in medical research [18,19]. Previous literature has reported an increase in the number of articles reporting power over time, and our analysis identifies a similar trend [16]. However, the large percentage of articles that omit power adequacy or admit inadequate power raises awareness about the potential pitfalls of underpowered studies [3,8,20]. Transparent reporting of power calculations is pivotal in accurately interpreting research findings and gauging study outcomes' reliability. Thus, it is preferred that articles discuss any potential limitations of their power analyses rather than omit them entirely when they are unsatisfactory. Orthopedic researchers and authors should strive to provide comprehensive power analyses to strengthen their conclusions.

In many subspecialties, there is a chronological increase in the number of published articles, a trend likely attributable to advancements in healthcare treatments or pressure on scholars to publish more research [21,22]. However, it is difficult to illustrate generalizable trends for the average number of statistical tests per paper within subspecialties. Some subspecialties, such as shoulder and elbow, show exponential growth across the three time intervals; conversely, others, such as the foot and ankle, remained relatively constant. Zhang et al. state that the average number of citations within subspecialties of orthopedic literature is contingent upon a subspecialty’s target audience, readership size, and impact on other non-orthopedic medical specialties [23]. The variance seen in subspecialty statistical test usage is likely attributable to similar factors [24]. There also appears to be no strong intra-specialty relationship between total publications and the average number of statistical tests. For instance, while arthroplasty had the highest number of publications, it ranked seventh out of 11 subspecialties for the average number of tests per paper. Overall, interspecialty variance calls for further and more nuanced analysis within orthopedic subspecialties.

Our findings have implications for both orthopedic practice and research. Clinicians engaging with orthopedic literature must cultivate a foundational understanding of statistical concepts to critically assess study findings. This knowledge enables clinicians to make informed decisions based on published evidence, promoting optimal patient care. Additionally, researchers must recognize the evolving landscape of statistical testing and leverage advanced statistical methods judiciously to enhance the precision and impact of their work. A study by Parsons et al. found that many conclusions drawn in orthopedic research are not properly justified by the results, emphasizing the importance of communication between statisticians, clinicians, and reviewers [8]. Collaboration between clinicians and researchers fosters a symbiotic relationship, facilitating the translation of research into improved orthopedic practices.

This study is not without limitations. Our analysis solely focused on trends within JBJS, whereas other journals have varying acceptance rates, authorship criteria, and other factors that may influence publication trends. As such, these results may not fully represent all orthopedic literature. Additionally, one reason the 30-year period studied in this article was chosen is to identify comprehensive trends in contemporary orthopedic research. However, with modern technological advancements, complex statistical analyses may become more efficient and accessible for researchers, leading to drastic changes in statistical test usage. Many elements of prior orthopedic literature trends were identified, and this study can serve as a baseline for where current orthopedic research stands and what improvements can be made. Future research can address these limitations by studying a larger time period with varying time intervals and by analyzing similar trends across multiple high-impact orthopedic journals.

In summary, investigating statistical testing trends within the JBJS illuminates the synergistic relationship between orthopedic research and quantitative analysis. As statistical methods continue to evolve, orthopedic professionals and researchers must uphold the principles of transparency and contextual understanding to ensure the credibility and applicability of their findings. Embracing the complexities of statistical testing adds depth to the discussion in orthopedics, driving the field toward evidence-based innovation and progress.

## Conclusions

As the field of orthopedic surgery continues growing, the usage of statistical tests within orthopedic literature concurrently increases. This study highlights the development of and provides a comprehensive framework for trends in statistical testing by analyzing articles published in JBJS over the last 30 years. Publications appropriately using statistical analysis help aid in the development of novel treatment options and improve the overall quality of patient care. Thus, physicians and researchers must maintain an understanding of foundational statistical concepts.
